# Effects of blood triglycerides on cardiovascular and all-cause mortality: a systematic review and meta-analysis of 61 prospective studies

**DOI:** 10.1186/1476-511X-12-159

**Published:** 2013-10-29

**Authors:** Jun Liu, Fang-Fang Zeng, Zhao-Min Liu, Cai-Xia Zhang, Wen-hua Ling, Yu-Ming Chen

**Affiliations:** 1 Guangdong Provincial Key Laboratory of Food, Nutrition, and Health, School of Public Health, Sun Yat-sen University, Guangzhou, Guangdong 510080, People’s Republic of China; 2 Department of Medicine and Therapeutics, The Chinese University of Hong Kong, Hong Kong, SAR, People’s Republic of China

**Keywords:** Triglycerides, Cardiovascular disease, All-cause, Mortality, Meta-analysis

## Abstract

The relationship of triglycerides (TG) to the risk of death remains uncertain. The aim of this study was to determine the associations between blood triglyceride levels and cardiovascular diseases (CVDs) mortality and all-cause mortality. Four databases were searched without language restriction for relevant studies: PubMed, ScienceDirect, EMBASE, and Google Scholar. All prospective cohort studies reporting an association between TG and CVDs or all-cause mortality published before July 2013 were included. Risk ratios (RRs) with 95% confidence intervals (CIs) were extracted and pooled according to TG categories, unit TG, and logarithm of TG using a random-effects model with inverse-variance weighting. We identified 61 eligible studies, containing 17,018 CVDs deaths in 726,030 participants and 58,419 all-cause deaths in 330,566 participants. Twelve and fourteen studies, respectively, reported the effects estimates of CVDs and total mortality by TG categories. Compared to the referent (90–149 mg/dL), the pooled RRs (95% CI) of CVDs mortality for the lowest (< 90 mg/dL), borderline-high (150–199 mg/dL), and high TG (≥ 200 mg/dL) groups were 0.83 (0.75 to 0.93), 1.15 (1.03 to 1.29), and 1.25 (1.05 to 1.50); for total mortality they were 0.94 (0.85 to 1.03), 1.09 (1.02 to 1.17), and 1.20 (1.04 to 1.38), respectively. The risks of CVDs and all-cause deaths were increased by 13% and 12% (p < 0.001) per 1-mmol/L TG increment in twenty-two and twenty-two studies reported RRs per unit TG, respectively. In conclusion, elevated blood TG levels were dose-dependently associated with higher risks of CVDs and all-cause mortality.

## Introduction

There is considerable evidence to suggest that triglycerides (TG) play a role in such adverse health conditions as heart disease, peripheral vascular disease, stroke, diabetes mellitus, metabolic syndrome, and cancer, which are common causes of death [1-3]. Several reviews and meta-analyses evaluated the association between blood TG and mortality. Hokanson et al. reported the summary crude RR for and CVDs death in the sensitive analysis of a meta-analysis assessing the association of TG with CVDs incidence [4], in which seventeen prospective studies of American and European participants were included. Two meta-analyses by Hokanson et al. updated the overall effect of TG on CVDs incidence but not for CVDs mortality [5,6]. The collaboration studies from the Asia Pacific Cohort Studies Collaboration (APCSC) suggested that elevated TG levels were strongly associated with an increased mortality of coronary heart disease (CHD) [7,8]. Another collaborative analysis including 10,269 participants from 7 studies in Europe, reported that higher triglyceride (≥ 1.7 versus. < 1.7 mmol/L) was associated with an increased risk of CVDs (but not all-cause) moratity in women and men [9]. Up to date, no study has assessed the overall effects of TG on CVDs death or all-cause death by including all eligible studies in the world. We conducted a systematic review and meta-analysis of prospective studies to evaluate the association between blood TG levels and CVDs mortality and all-cause mortality.

## Methods

### Literature search strategies

Following the methodology advocated in the Meta-Analysis of Observational Studies in Epidemiology (MOOSE) guidelines [10] and without language restrictions, we carried out a systematic search of four electronic databases, PubMed (1966 to July 2013), ScienceDirect (1960 to July 2013), EMBASE (1980 to July 2013), and Google Scholar (http://scholar.google.com) without language restrictions using the following search terms: (i) triglyceride* or triacylglycerol* or TG or lipids; (ii) blood or serum or plasma or circulating; (iii) cohort* or longitudinal or follow-up or prospective or relative risk* or hazard ratio*; (iv) death* or mortali*; and (v) (i) AND (ii) AND (iii) AND (iv). We also searched the reference lists of original and review articles to identify more studies. Related studies generated by PubMed were also retrieved. If necessary, we contacted authors for additional data.

### Inclusion and exclusion criteria

We included studies that met all of the following criteria: (1) prospective cohort design in a general population; (2) the exposure of interest included TG; (3) the outcome of interest included all-cause mortality or CVDs mortality; and (4) the relative risks (RRs) or hazard ratios (HRs) with their corresponding 95% confidence intervals (CIs) (or sufficient data to estimate them) were reported according to at least three TG categories, or by unit of TG, or by logarithmically transformed TG. We excluded studies that were focused on patients with one or several specific types of disease, such as diabetes, CVDs, dyslipidemia, or cancer. If multiple published reports from the same population resource or overlapping datasets were available, we included only the most relevant article with the largest dataset.

### Data extraction and quality assessment

Two epidemiologists (JL and FFZ) independently assessed the eligible studies, collected information, and assessed the quality of the data. Any discrepancies were resolved through discussion. All relevant information was recorded, included the article title, the first author’s name, publication year, country in which the study was performed, population inclusion and exclusion criteria, sample size at baseline, mean (range) duration of follow-up, mean (range) age at baseline, proportion of men, geographic location, type of blood sample (serum, plasma, or other), fasting status (yes, no, or other), whether there was an adjustment for total cholesterol (TC) or not, and risk estimate (RR or HR) with corresponding 95% CI after controlling for both the minimally and the maximally adjusted number of covariates.

The Newcastle-Ottawa Quality Assessment Scale (NOS) was applied to assess the quality of each included study [11]. The NOS was designed to assesses the quality of cohort studies in terms of their selection of participants (four criteria), degree of comparability between study groups (one criterion), and assessment of outcomes (three criteria). Total scores range from 1 to 9, with 9 being the maximum.

### Statistical analysis

We conducted three separate meta-analyses for the risk estimates on the basis of the TG categories, unit TG, and logarithm of TG. For the category data, we standardized and grouped the TG levels with unit mg/dL into the four categories used in the Adult Treatment Panel III (ATP III) cholesterol guidelines [12]: lowest group (< 90 mg/dL), intermediate group (90–149 mg/dL), borderline hypertriglyceridemia group (150–199 mg/dL), and hypertriglyceridemia group (≥ 200 mg/dL). The intermediate group was used as the referent. As the highest category in each individual study was open-ended, we assigned the category a value equal to 50% of the width of its lower boundary. If one or more group in a single study fell into the same category, we pooled these risk estimates first. We analyzed the dose–response relationships using the method proposed by Greenland et al. [13,14]. Study-specific slopes (linear trends) were calculated from the natural log of the RRs across various exposure levels, correlated with their corresponding TG levels. The original dose groups were used in the dose–response relationship analysis. For the studies reporting the risk estimates by unit TG, the RRs were standardized to a 1-mmol/L increase in TG according to the following formulas.

$$\begin{array}{l} \;RR_{\mathrm{st}}=\text{exp}\left(\ln RR_{o}/k\right),\\ 95\%\;\mathrm{CI}=\text{exp}\left(\ln RR_{o}/k\pm 1.96SE_{o}/k\right) \end{array}$$

$$\;SE_{o}=\left(\ln RR_{o\_\mathrm{ul}}–{\mathrm{lnRR}}_{o\_\mathrm{ll}}\right)/\left(2*1.92\right)$$

Where, *RR*_*st*_ is the standardized RR; *RR*_*o*_ is derived from the confidence intervals provided in each study; *ul* is the upper limit; *ll* is the lower limit; and *k* refers to the per *k*-mmol/L increase in TG levels in the original study.

For the studies reporting the risk estimates by logarithmically transformed TG, we pooled the risk estimates per unit of log of TG (mmol/L).

The DerSimonian and Laird random-effects model of inverse variance methods was used to estimate the pooled risks and 95% CIs [15]. RRs were used for all of the risk estimates including RRs and HRs. Unless otherwise stated, we used the most fully adjusted RRs from each study. Statistical heterogeneity was tested with the *I*^2^ statistic; *I*^2^ values of 25% to < 50%, 50% to < 75%, and ≥ 75% were considered to represent small, medium, and large degree of inconsistency, respectively [16]. The Begg’s and Egger’s regression tests were used to detect any publication bias [17,18] and a trim-and-fill method was used to adjust for any suspected publication bias once identified [19].

Subgroup analyses were carried out to identify possible sources of heterogeneity and to check for the potential effects of age (≥ 50 or not), gender (men/women), duration of follow-up (≥15 or not), sample size (≥ 4000 or not), fasting status (Yes or No), geographic location (Europe/America or Asia-Pacific), blood sample (serum/plasma), free CVDs history at baseline (Yes or No), study quality (> 6 score or not), and adjustment for TC (Yes or No) on the relationship between TG and the outcomes. We also performed a meta-regression to explore the independent influences of these factors on heterogeneity and then sensitivity analysis on the main effects by excluding all of the studies one by one to evaluate whether any single or group of studies might markedly affect the results. We also compared the pooled estimates using RRs adjusted for the minimal and maximal number of covariates in the studies that reported both. Finally, using the methods proposed by Mantel and Haenszel, we performed the chi-square test for heterogeneity to examine differences in pooled RRs [20]. All of these tests were two-sided and statistical significance was defined as *P <* 0.05. The analyses were performed with the Stata statistical software version 11.0 (College Station, TX, USA).

## Results

### Study characteristics

**Figure 1:**
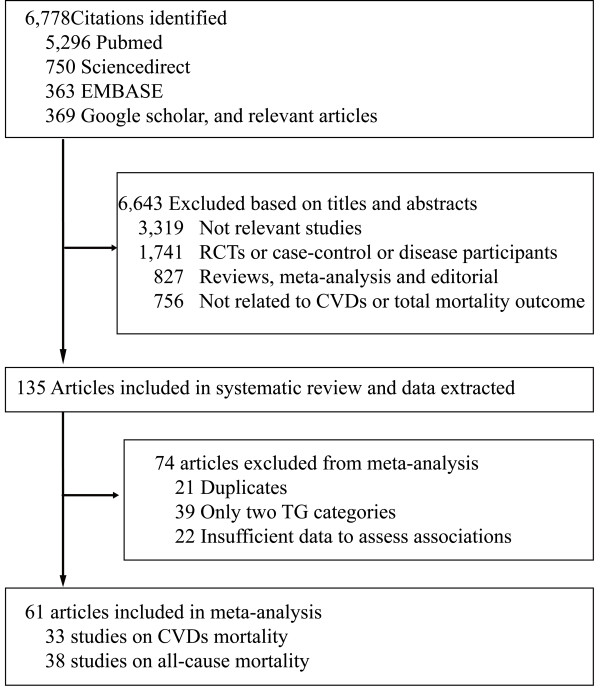
**Flow diagram of study selection.** TG: Triglycerides; CVDs: Cardiovascular diseases

Figure 1 outlines our search and selection process, and illustrates the detailed population selection criteria in the original studies. A total of 61 studies included in the present meta-analysis were pooled according to CVDs mortality and all-cause mortality. They were all published in English with the exception of one published in Norwegian study [21]. The features of these studies are summarized in Additional file 1: Table S1. Thirty-three studies reported data on CVDs mortality; together recording 17,018 CVDs deaths in 726,030 participants; and 38 studies reported data on all-cause mortality, together recording 58,419 deaths in 330,566 participants. The median age at the recruitment was 48.0 and median duration of follow-up was 12.0 years.

### Overall effects of triglycerides on CVDs and all-cause mortality

**Figure 2:**
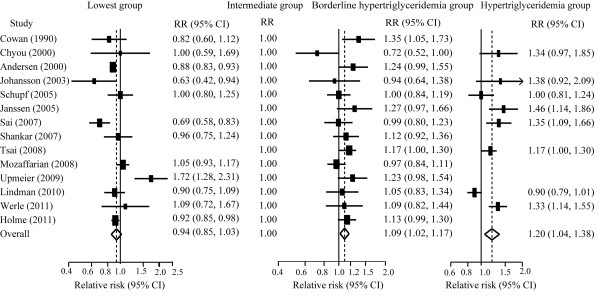
**Pooled estimate of RR with 95% CI of all-cause mortality for category analysis.** Squares indicate the adjusted relative risk (RR) and horizontal lines indicate the 95% confidence interval (CI). Triglycerides (TG) groups: lowest (<90 mg/dL), intermediate (90–149 mg/dL, the referent), borderline high (150–199 mg/dL), and high (≥ 200 mg/dL).

**Figure 3:**
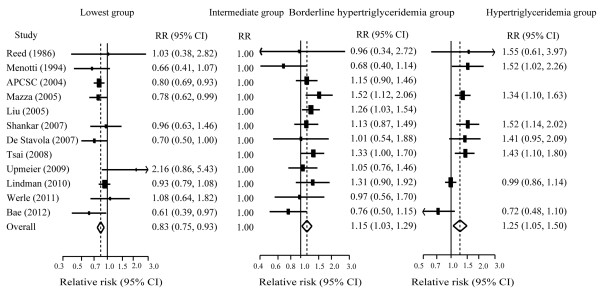
**Pooled estimate of RR with 95% CI of CVDs mortality for categorical analysis.** Squares indicate the adjusted relative risk (RR) and horizontal lines indicate the 95% confidence interval (CI). Triglycerides (TG) groups: lowest (<90 mg/dL), intermediate (90–149 mg/dL, the referent), borderline high (150–199 mg/dL), and high (≥ 200 mg/dL). CVDs: cardiovascular diseases.

Twelve and fourteen studies, respectively, reported the effects estimates of CVDs and total mortality by TG categories. When compared to individuals with TG level (90 mg/dL-149 mg/dL), those with TG level less than 90 mg/dL had no significant risk of all-cause mortality, but had less risk of CVDs moratlity, those with borderline hypertriglyceridemia (150 mg/dl-199 mg/dl) hypertriglyceridemia (≥ 200 mg/dL) had a greater risk of CVDs and all-cause mortality (Figure 2 and Figure 3). The dose–response analysis showed that the risk of CVDs and total mortality was increased by 13% (8%-19%) and 10% (6%-14%) per 1-mmol/L TG increment (Additional file 2: Figure S2).

**Figure 4:**
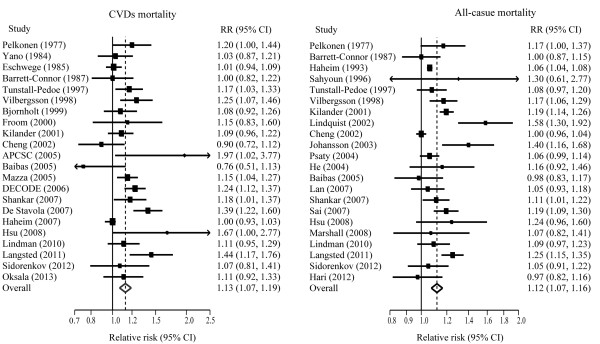
**Pooled estimate of RR with 95% CI of CVDs and all-cause mortality for a 1-mmol/L increase of TG.** Squares indicate the adjusted relative risk (RR) and horizontal lines indicate the 95% confidence interval (CI). TG: Triglycerides; CVDs: cardiovascular diseases.

**Figure 5:**
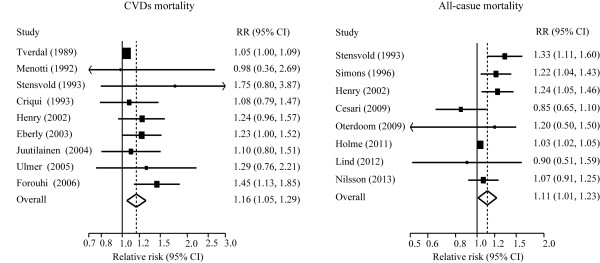
**Pooled estimate of RR and 95% CI of CVDs and all-cause mortality for a 1-ln (mmol/L) increase of TG.** TG: Triglycerides; CVDs: cardiovascular diseases; CI, confidence interval; RR, relative risk.

Twenty-two and twenty-two studies, respectively, were included in the analysis of risk for CVDs and total mortality by unit TG, and nine and eight studies, respectively, reported the results of CVDs and total mortality by log TG. Figure 4 and Figure 5 show overall association of TG with the risks of CVDs and all-cause mortality.

The heterogeneity across studies varied from null to large (*I*^*2*^ = 0%–75.9%) and no obvious publication biases were observed (*P* = 0.150 to 1.00) in the foregoing analyses (Table 1).

**Table 1:** Pooled relative risks of CVDs and all-cause mortality by blood TG levels

	**N**	**Overall effects**		***I***^**2**^	***P *** **for heterogeneity**	**Begg’s test**	**Influence analyses**	
		**RR (95% CIs) **^**†**^	***P***	**(%)**		**(P value)**	**Minimum**	**Maximum**
Cardiovascular disease mortality								
TG groups, mg/dl								
<90	10	0.83 (0.75, 0.93)	0.001	21.6	0.244	0.592	0.80 (0.71, 0.91)	0.85 (0.76, 0.94)
90-150	12	1.00 (referent)						
150-200	12	1.15 (1.03, 1.29)	0.015	22.5	0.222	0.150	1.12 (1.00, 1.27)	1.19 (1.08, 1.32)
>200	8	1.25 (1.05, 1.50)	0.013	66.2	0.004	0.711	1.22 (1.00, 1.49)	1.33 (1.13, 1.57)
Per 1 mmol/L TG	22	1.13 (1.07, 1.19)	0.000	65.9	0.000	1.000	1.11 (1.06, 1.17)	1.14 (1.08, 1.20)
Per 1ln (mmol/L TG)	9	1.16 (1.05, 1.29)	0.004	32.3	0.160	0.754	1.06 (1.02,1.11)	1.24 (1.11, 1.39)
All-cause mortality								
TG groups, mg/dl								
<90	12	0.94 (0.85, 1.03)	0.150	71.1	0.000	0.732	0.90 (0.83, 0.97)	0.96 (0.84, 1.06)
90-150	14	1.00 (referent)						
150-200	14	1.09 (1.02, 1.17)	0.011	33.3	0.108	1.000	1.08 (1.00, 1.16)	1.11 (1.04, 1.19)
00A0 > 200	8	1.20 (1.04, 1.38)	0.011	75.4	0.000	0.711	1.17 (1.01, 1.35)	1.25 (1.14, 1.37)
Per 1 mmol/L TG	22	1.12 (1.07, 1.16)	0.000	75.9	0.000	0.866	1.11 (1.07, 1.15)	1.13 (1.08, 1.18)
Per 1 ln (mmol/L TG)	8	1.11 (1.01, 1.23)	0.030	62.4	0.010	0.711	1.08 (0.99, 1.18)	1.14 (1.02, 1.28)

† Random-effects model.

RR, relative risk; CI, confidence interval; TG: triglycerides; CVDs: cardiovascular diseases.

### Sensitivity and subgroup analyses

The subgroup analyses showed that the pooled RRs were significantly higher in men than in women and higher in studies with longer follow-up durations for the risk of both CVDs and total mortality; and tended to be higher for CVDs mortality in the studies that adjusted for TC (RR: 1.09 versus 1.21, *P* = 0.078). Similar results were observed in the meta-regression analyses. No other significant between-group heterogeneities were observed in the subgroup analyses (Tables 2 and 3) or in the meta-regression (Additional file 3: Table S3).

**Table 2:** Pooled relative risks of CVDs mortality for categorical and continuous analyses in subgroups

**Sub-groups**	**Triglyceride group (mg/dl)**								**Per 1 mmol/L TG**	
	**<90**		**90-146**		**150-199**		**≥200**			
	N*	**RR (95% CI)**	N*	**RR†**	N*	**RR (95% CI)**	N*	**RR (95% CI)**	N*	**RR (95% CI)**
*Age (year)*										
<50	3	0.84 (0.74, 0.96)	4	1.00	4	1.21 (1.06, 1.40)	3	1.20 (0.94, 1.53)	10	1.11 (1.01, 1.22)
≥50	7	0.85 (0.68, 1.05)	8	1.00	8	1.08 (0.90, 1.30)	5	1.30 (0.99, 1.69)	12	1.15 (1.09, 1.22)
P value		0.956				0.338		0.664		0.488
*Gender*										
Male	5	0.86 (0.67, 1.09)	5	1.00	5	1.06 (0.89, 1.30)	5	1.31 (1.12, 1.15)	18	1.10 (1.04, 1.15)
Female	3	0.76 (0.64, 0.90)	3	1.00	3	1.37 (1.09, 1.71)	2	2.08 (1.44, 3.00)	9	1.40 (1.22, 1.59)
Mixed	4	1.01 (0.65, 1.56)	6	1.00	6	1.14 (1.10, 1.31)	4	1.26 (0.99, 1.60)	4	1.16 (1.08, 1.25)
P value		0.416				0.100		0.023		0.001
*Follow-up (year)*										
<12	6	0.81 (0.71, 0.91)	6	1.00	6	1.01 (0.84, 1.19)	4	1.25 (0.84, 1.84)	11	1.14 (1.06, 1.22)
≥12	4	0.79 (0.61, 1.02)	4	1.00	6	1.27 (1.13, 1.44)	4	1.25 (1.01, 1.54)	11	1.11 (1.02, 1.21)
P value		0.473				0.025		0.992		0.694
*Fast status*										
Fast	6	0.89 (0.71, 1.12)	7	1.00	7	1.15 (0.98, 1.35)	4	1.42 (1.22, 1.64)	10	1.10 (1.04, 1.17)
Nonfast	3	1.00 (0.65, 1.54)	4	1.00	4	1.14 (0.85, 1.46)	4	1.11 (0.85, 1.46)	7	1.12 (1.01, 1.23)
Others^§^	1	0.80 (0.69, 0.93)	1	1.00	1	1.15 (0.90, 1.45)	0	-	5	1.19 (1.00, 1.41)
P value		0.646				0.843		0.122		0.821
*Geographic location*										
Europe/America	7	0.86 (0.73, 1.01)	8	1.00	8	1.17 (1.00, 1.37)	5	1.22 (1.02, 1.55)	18	1.11 (1.05, 1.17)
Asia-Pacific	3	0.80 (0.70, 0.92)	4	1.00	4	1.20 (0.93, 1.35)	3	1.20 (0.82, 1.77)	4	1.26 (1.05, 1.52)
P value		0.509				0.729		0.847		0.205
*Quality score*										
<7	4	0.76 (0.65, 0.90)	5	1.00	5	1.01 (0.80, 1.29)	4	1.24 (0.91, 1.68)	8	1.16 (1.04, 1.29)
7-9	6	0.92 (0.77, 1.05)	7	1.00	7	1.23 (1.09, 1.38)	4	1.25 (0.98, 1.60)	14	1.11 (1.04, 1.19)
P value		0.100				0.167		0.961		0.509
*Sample size*										
<4000	7	0.84 (0.70, 1.02)	7	1.00	7	1.10 (0.91, 1.32)	4	1.49 (1.21, 1.80)	10	1.14 (1.06, 1.22)
≥4000	3	0.83 (0.71, 0.97)	5	1.00	5	1.18 (1.02, 1.38)	4	1.12 (0.84, 1.45)	12	1.13 (1.04, 1.22)
P value		0.910				0.541		0.100		0.883
*Free of CVDs at baseline*										
Yes	6	0.85 (0.76, 0.96)	7	1.00	7	1.12 (0.92, 1.36)	6	1.20 (0.96, 1.50)	13	1.11 (1.04, 1.20)
No	4	0.88 (0.67, 1.56)	5	1.00	5	1.16 (1.00, 1.34)	2	1.43 (1.16, 1.76)	9	1.16 (1.09, 1.24)
P value		0.832				0.811		0.265		0.371
*Adjustment*										
Minimally	4	0.72 (0.55, 0.94)	5	1.00	5	1.44 (1.16, 1.78)	4	1.52 (1.14, 2.02)	7	1.15 (1.08, 1.22)
Maximally	4	0.87 (0.77, 0.98)	5	1.00	5	1.24 (1.05, 1.46)	4	1.29 (1.01, 1.65)	7	1.05 (0.99, 1.11)
P value		0.207				0.271		0.398		0.033
*Adjustment for TC*										
Yes	4	0.85 (0.77, 0.94)	4	1.00	4	1.24 (1.07, 1.43)	3	1.27 (0.94, 1.71)	14	1.09 (1.03, 1.16)
No	6	0.82 (0.61, 1.09)	8	1.00	8	1.06 (0.89, 1.26)	5	1.25 (0.97, 1.61)	8	1.21 (1.10, 1.34)
P value		0.810				0.169		0.927		0.078
*Adjustment for HDL*										
Yes	3	0.81 (0.72, 0.91)	3	1.00	3	1.23 (1.04, 1.47)	1	1.52 (1.14, 2.02)	3	1.10 (1.03, 1.19)
No	7	0.84 (0.68, 1.04)	9	1.00	9	1.09 (0.94, 1.27)	7	1.21 (1.00, 1.47)	19	1.13 (1.06, 1.20)
P value		0.734				0.313		0.200		0.679

*The number of cohorts.

†Reference group.

§Others included mixed and unknown ones.

RR, relative risk; CI, confidence interval; CVDs: cardiovascular diseases; HDL: high-density lipoprotein.

TG: triglycerides; TC: total cholesterol.

**Table 3:** Pooled relative risks of all-cause mortality for categorical and continuous analyses in subgroups

**Sub-groups**	**Triglyceride group (mg/dl)**								**Per 1 mmol/L TG**	
	**<90**		**90-146**		**150-199**		**≥200**			
	N*	**RR (95% CI)**	N*	**RR†**	N*	**RR (95% CI)**	N*	**RR (95% CI)**	N*	**RR (95% CI)**
*Age (year)*										
<50	3	0.88 (0.78, 1.00)	5	1.00	5	1.14 (1.05, 1.24)	5	1.20 (0.99, 1.46)	8	1.13 (1.05, 1.21)
≥50	9	0.97 (0.85, 1.11)	9	1.00	9	1.07 (0.97, 1.18)	3	1.20 (0.97, 1.48)	14	1.12 (1.07, 1.17)
P value		0.311				0.329		0.982		0.845
*Gender*										
Male	8	0.95 (0.84, 1.09)	9	1	9	1.12 (1.02, 1.22)	5	1.23 (1.09, 1.38)	14	1.09 (1.04, 1.13)
Female	6	0.91 (0.84, 0.99)	6	1	6	1.17 (0.99, 1.39)	2	1.01 (0.59, 1.74)	10	1.24 (1.15, 1.32)
Mixed	3	1.00 (0.85, 1.17)	4	1	4	1.11 (1.01, 1.21)	3	1.17 (1.01, 1.35)	6	1.08 (1.03, 1.13)
P value		0.577				0.621		0.491		0.001
*Follow-up (year)*										
<15	6	0.89 (0.84, 0.94)	7	1.00	7	1.11 (0.97, 1.26)	4	1.26 (1.06, 1.51)	14	1.05 (1.02, 1.07)
≥15	6	0.94 (0.79, 1.11)	7	1.00	7	1.09 (1.02, 1.16)	4	1.15 (0.93, 1.41)	8	1.22 (1.16, 1.29)
P value		0.569				0.773		0.490		0.000
*Fast status*										
Fast	8	1.01 (0.86, 1.20)	11	1.00	11	1.08 (0.98, 1.18)	5	1.27 (1.09, 1.48)	15	1.12 (1.06, 1.19)
Nonfast	2	0.91 (0.86, 0.98)	3	1.00	3	1.14 (1.04, 1.24)	3	1.11 (0.88, 1.40)	6	1.11 (1.03, 1.19)
Others^§^	2	0.79 (0.63, 0.99)	2	1.00	2	1.11 (0.89, 1.38)	0	-	1	1.19 (1.09, 1.30)
P value		0.258				0.381		0.335		0.740
*Geographic location*										
Europe/America	10	0.96 (0.87, 1.06)	11	1.00	11	1.09 (1.00,1.19)	6	1.19 (0.98, 1.45)	16	1.12 (1.06, 1.17)
Asia-Pacific	2	0.81 (0.59, 1.11)	3	1.00	3	1.12 (1.02, 1.23)	2	1.22 (1.08, 1.39)	6	1.13 (1.08, 1.20)
P value		0.302				0.633		0.813		0.640
*Quality score*										
<7	5	0.90 (0.86, 0.94)	7	1.00	7	1.15 (1.08, 1.24)	4	1.22 (1.07, 1.40)	10	1.13 (1.08, 1.18)
7-9	7	0.95 (0.77, 1.17)	7	1.00	7	1.03 (0.91, 1.17)	4	1.19 (0.91, 1.57)	12	1.11 (1.05, 1.18)
P value		0.594				0.116		0.869		0.707
*Sample size*										
<4000	7	0.97 (0.75, 1.25)	7	1.00	7	1.03 (0.92, 1.15)	4	1.22 (1.03, 1.45)	13	1.14 (1.07, 1.22)
≥4000	5	0.92 (0.86, 0.96)	7	1.00	7	1.14 (1.04, 1.24)	4	1.18 (0.95, 1.45)	9	1.09 (1.04, 1.14)
P value		0.729				0.155		0.778		0.190
*Free of CVDs at baseline*										
Yes	5	0.94 (0.85, 1.03)	6	1.00	6	1.07 (0.99, 1.16)	5	1.24 (0.99, 1.56)	13	1.10 (1.04, 1.16)
No	7	0.97 (0.80, 1.16)	8	1.00	8	1.10 (0.99, 1.23)	3	1.14 (1.00, 1.30)	9	1.13 (1.07, 1.20)
P value		0.711				0.663		0.519		0.480
*Adjustment*										
Minimally	9	0.86 (0.72, 1.03)	10	1.00	10	1.14 (0.99, 1.30)	7	1.38 (1.13, 1.67)	8	1.12 (1.01, 1.24)
Maximally	9	0.94 (0.80, 1.11)	10	1.00	10	1.07 (0.99, 1.17)	7	1.18 (1.01, 1.38)	8	1.07 (1.01, 1.13)
P value		0.466				0.487		0.222		0.432
*Adjustment for TC*										
Yes	5	0.84 (0.7, 0.99)	5	1.00	5	1.03 (0.93, 1.14)	2	1.10 (0.88, 1.38)	11	1.10 (1.05, 1.15)
No	7	1.00 (0.89, 1.12)	9	1.00	9	1.12 (1.02, 1.24)	8	1.27 (1.16, 1.40)	11	1.14 (1.07, 1.21)
P value		0.103				0.225		0.239		0.382
*Adjustment for HDL*										
Yes	2	1.03 (0.93, 1.14)	2	1.00	2	1.02 (0.89, 1.17)	1	1.35 (1.09, 1.66)	4	1.07 (1.04, 1.11)
No	10	0.92 (0.82, 1.02)	12	1.00	12	1.11 (1.03, 1.20)	8	1.14 (0.93, 1.39)	18	1.12 (1.07, 1.17)
P value		0.115				0.296		0.250		0.132

*The number of cohorts.

†Reference group.

§Others included mixed and unknown ones.

RR, relative risk; CI, confidence interval; CVDs: cardiovascular diseases; HDL: high-density lipoprotein.

TG: triglycerides; TC: total cholesterol.

Sensitivity and influence analyses were conducted on the pooled risks and the associations of the lowest group tended to be vague when one studies [22] was excluded for total mortality. No single study significantly influenced the pooled estimates risks by unit of TG or for any other of TG categories group. The pooled maximally adjusted RR of CVDs mortality was markedly lower than that of its minimally adjusted RR in the continuous analysis (RR =1.05 versus 1.15, *P* = 0.033), but not in the categorical analysis (*P*: 0.207 to 0.398). No significant differences were observed in the all-cause mortality data (*P*: 0.222 to 0.487) (Tables 2 and 3).

## Discussion

We quantitatively assessed the association between blood triglyceride levels at baseline and CVDs mortality in 33 studies with 17,018 cases among 726,030 participants, as well as all-cause mortality in 38 studies with 58,419 cases among 360,566 participants. Compared to the referent, the risks of CVDs and all-cause mortality were increased by 15.0% and 9.0% in the borderline hypertriglyceridemia group, and 25% and 20% in the hypertriglyceridemia group, respectively. Overall, the risks of CVDs and all-cause death were increased by 13% and 12% per 1-mmol/L increase in TG level.

These findings were robust. We incorporated a large number of participants and deaths (a total of 17,018 CVDs deaths in 726,030 participants and 58,419 all-cause deaths in 330,566 participants), which improved the statistical power for detecting potential associations. Additionally, the studies included in our sample were carried out worldwide, with participants from the America, Europe, Asia, and Australia, thus enhancing generalizability. Furthermore, only prospective cohort studies were considered. The participants in 33 of the studies had no history of CVDs at baseline and the follow-up duration in 44 was more than 10 years, largely eliminating the possibility of reverse causation relationship. Participants were also selected from the general population, thus reducing potential selection bias. In addition, we used the Newcastle-Ottawa Scale to evaluate the quality of studies and most of the studies (50 studies) included in this meta-analysis were high-quality (score >6, full score = 9). Finally, there was a large degree of consistency between the continuous and categorical analyses.

We observed that elevated TG levels were associated with an increased risk of CVDs mortality echoing the findings of several other meta-analyses of prospective studies demonstrating a relationship between higher TG levels and an increased risk of cardiovascular events [4,5,23]. In 1996, Hokanson et al. [4] performed a meta-analysis comprising 46,413 men and 10,864 women from the USA and Europe. Their summary crude RRs for fatal CVDs from seven prospective studies were 1.24 in men and 1.84 in women per a 1-mmol/L increase. In the present meta-analysis, the RRs of CVDs mortality was lower. However, the discrepancy was unlikely to be caused by geographic variations as we observed similar results in the European and American and Asia-Pacific samples in the present study. Hypertriglyceridemia is commonly associated with diabetes, obesity, hypertension, and smoking, which are independent risk factors for CHD [24,25]. The pooled crude RR in the study by Hokanson et al. might be confounded by these factors as the adjusted overall RR of fatal and non-fatal CVDs for women declined from the crude value of 1.76 to 1.37 [4]. The subgroup analysis of the seven studies that reported both maximally and minimally adjusted data demonstrated that the overall RRs were significantly lower for the maximally adjusted original data, than that for the minimally adjusted. In the present meta-analysis, the data in the majority of the studies were adjusted for common potential confounders, such as age, gender, blood pressure, BMI, diabetes, smoking and TC, and would thus have better validity than unadjusted data.

We also found that an association between elevated TG levels and an increased risk of all-cause mortality. In fact, TG levels were found to have a similar predictive power for CVDs and all-cause mortality. It was thus reasonable to hypothesize that there was a positive relationship between TG and non-CVDs death. Prospective studies have found that elevated TG levels increases the risk of non-CVDs mortality [26]. A collaborative study of metabolic syndrome and cancer (Me-Can) involving 514,097 participants with a 13.4-year follow-up, demonstrated a positive association between serum TG and risk of cancer overall and at several sites. The RR (95% CI) for the top quintile versus the bottom quintile of triglycerides for overall cancer was 1.16 (1.06 to 1.26) in men and 1.15 (1.05 to 1.27) in women [3]. Previous studies have also reported that elevated TG levels increased the risks of other deaths, such as from kidney disease [27,28] or suicide [29]. Hence, non-CVDs mortality may contribute to the relative risk of all-cause mortality.

There are several possible explanations for these findings. Firstly, TG is associated with atherogenic remnant particles, which have a high uptake into macrophages leading to foam cell formation. Furthermore, triglyceride-rich lipoproteins and their remnants promote inflammation and increase the expression of coagulation factors or leukocyte adhesion molecules [2]. An increased number of atherogenic particles may thus adversely influence CVDs risk. Secondly, hypertriglyceridemia is associated with the development of oxidative stress and reactive oxygen species (ROS) [30]. ROS directly influences cell proliferation and apoptosis and modulates DNA methylation patterns; and thus may contribute to the multistage carcinogenesis process [31]. Additionally, increased oxidative stress in fat has been shown to be an important pathogenic pathway in metabolic syndrome. In addition, elevated triglyceride-rich lipoproteins may adversely influence the risk of chronic kidney diseases, as triglyceride-rich apolipoprotein B-containing lipoproteins promote the progression of renal insufficiency [32]. Moreover, oxidative stress and endothelial dysfunction may cause atherosclerosis-related kidney damage in older people [33]. Finally, hypertriglyceridemia has been associated with increased cortisol levels in response to stress and symptoms of depression, which may lead to an increased risk of suicide [29].

In the present study, we found that elevated blood TG was associated with greater total death and CVDs death risk in women than in men, which was consistent with prior studies [4,5]. A collaborative study and a large prospective study also showed that TC and HDL-c were more predictive of CVDs mortality in women than in men [34,35]. However, the mechanism remains unclear and further studies are needed to clarify this issue. The subgroup analysis in the present study showed that studies with longer follow-up periods had significantly higher risk estimates for mortality, possibly due to the greater cumulative risks associated with longer TG exposure.

This meta-analysis has several limitations. First, there was considerable heterogeneity among the studies included with analysis showing that the major sources of heterogeneity to be gender, adjustment for TC, and follow-up duration. Second, 28 studies that included participants with a prior history of CVDs at baseline were not excluded. Previous studies have reported that excluding participants with a history of CVDs at baseline did not substantially change the risks of CVDs mortality [36]. In addition, subgroup analysis suggested that the overall RRs for all-cause and CVDs mortality in relation to TG were not markedly influenced by prior CVDs history. Third, elevated TG levels were accompanied by low HDL-c and high TC, both of which have been associated with a higher CVDs risk [1,2]. In the present meta-analysis, subgroup analysis observed that the effect sizes were significantly decreased after adjustment for TC although the positive relationship remains significant. The overall effect estimates were unlikely to be biased by TC since most of original studies (45/61) reported TC-adjusted RRs, but it tended to be overestimated because few (10/61) studies reported HDL-adjusted RRs. Fourth, the studies included in the present meta-analysis were mainly based on a one-time measurement of baseline blood TG (five studies adjusted for regression dilution bias), which may underestimate the associations due to regression dilution effects. Finally, the relatively small number of studies that included categorical analysis limited the ability to detect heterogeneity in the subgroup analysis.

In conclusion, elevated blood TG levels were dose-dependently associated with a greater risk of both CVDs and all-cause mortality. The findings of this meta-analysis suggest that controlling TG can help to prevent CVDs and other causes of death.

## Abbreviations

TG: Triglycerides; CVDs: Cardiovascular diseases; APCSC: Asia pacific cohort studies collaboration; CHD: Coronary heart disease; TC: Total cholesterol; HDL-C: High-density lipoprotein; BMI: Body mass index; ROS: Reactive oxygen species.

## Competing interests

The authors declare that they have no competing interests.

## Authors’ contributions

JL and FFZ contributed to the collation of data and their critical review, and manuscript writing. JL performed the meta-analysis. YMC concepted and designed the study, and critically revised the paper and had responsibility for its final content. ZML, CXZ and WHL critically revised the paper. All authors have read and approved the final manuscript.

## Supplementary Material

Additional file 1: Table S1 Characteristics of the original studies and the study population. Click here for file

Additional file 2: Figure S1 Dose–response relationships between TG and risk of CVDs and all-cause mortality. The dots represent the RRs corresponding to TG levels in each individual study. The area of the dots is inversely proportional to the logarithm of the RR variance. The three curves are the RR estimates and their 95% CIs according to the dose–response model. CVDs, cardiovascular diseases; CI, confidence interval; RR, relative risk; TG, triglycerides. Click here for file

Additional file 3: Table S3 Meta regression analysis of TG and CVDs and all-cause mortality for continuous analysis. Click here for file
