# Supplementary material for: Effects of blood triglycerides on cardiovascular and all-cause mortality: a systematic review and meta-analysis of 61 prospective studies
Source: Lipids Health Dis. 2013 Oct 29;12:159. doi: 10.1186/1476-511X-12-159 (PMC4231478; doi:10.1186/1476-511X-12-159)
Supplement: Additional file 1: Table S1 — Characteristics of the original studies and the study population. [file 1476-511X-12-159-S1.doc]

**Additional file 1 Characteristics of the original studies and the study population**

| Source | Country | No. of  subjects | Age^†^  (y) | Gender  Male% | Follow  -up (y) ^‡^ | No. of  CVDs death | No. of  total death | Blood  sample | Fast  status | Quality Score^§^ | Adjusted for  ABCDEFG+n^¶^ | TG variable^*^ | |
| --- | --- | --- | --- | --- | --- | --- | --- | --- | --- | --- | --- | --- | --- |
|  |  |  |  |  |  |  |  |  |  |  |  | Continuous | Category |
| Pelkonen (1977) | Finland | 1,648 | 51.5 | 100 | 7.0 | 75 | 129 | Serum | No | 6 | +1 | √ |  |
| Yano (1984) | USA | 7,705 | 54.4 | 100 | 10.0 | 139 |  | Serum | No | 7 | +6 | √ |  |
| Eschwege (1985) | France | 7,164 | 46.0 | 100 | 11.2 | 126 |  | Serum | Yes | 6 | +2 | √ |  |
| Reed (1986) | USA | 1,545 | 58.0 | 100 | 10.0 | 32 |  | Serum | Yes | 8 | +0 |  | √ |
| Barrett-Connor (1987) | USA | 1,589 | 50.0 | 100 | 12.0 | 144 | 349 | Plasma | Yes | 8 | +1 | √ |  |
| Tverdal (1989) | Norway | 37,546 | 45.0 | 100 | 9.0 | 369 |  | Serum | No | 8 | +1 | √^＃＃^ |  |
| Cowan (1990) | USA | 5,229 | 59.5 | 53 | 8.4 |  | 457 | Plasma | Yes | 5 | +0 |  | √ |
| Menotti (1992) | Italian | 3,395 | 55.1 | 100 | 10.0 | 107 |  | Serum | Yes | 7 | +8 | √^＃＃^ |  |
| Haheim (1993) | Norway | 14,403 | 45.4 | 100 | 12.0 |  | 740 | Serum | No | 8 | +2 | √ |  |
| Criqui (1993) | USA | 7,505 | 58.2 | 55 | 12.2 | 201 |  | Plasma | Yes | 7 | +3 | √^＃＃^ |  |
| Stensvold (1993) | Norway | 49,593 | 43.0 | 50 | 14.6 | 108 | 931 | Serum | No | 7 | +3 | √^＃＃^ |  |
| Menotti (1994) | Italian | 3,007 | 55.0 | 100 | 10.0 | 107 |  | Serum | Yes | 6 | +0 |  | √ |
| Sahyoun (1996) | USA | 287 | 74.6 | 35 | 12.0 |  | 81 | Serum | Yes | 5 | +5 | √ |  |
| Simons (1996) | Australia | 1569 | 69.2 | 0 | 5.0 |  | 184 | Serum | Yes | 6 | +8 | √^＃＃^ |  |
| Tunstall-Pedoe (1997) | UK | 11,629 | 50.0 | 49 | 7.6 | 206 | 591 | Serum | Yes | 6 | +0 | √ |  |
| Vilbergsson (1998) | Iceland | 18,887 | 52.8 | 48 | 17.0 | 1,458 | 4,380 | Serum | Yes | 7 | +1 | √ |  |
| Bjornholt (1999) | Norway | 1,973 | 50.0 | 100 | 22.0 | 483 |  | Serum | Yes | 8 | +4 | √ |  |
| Andersen (2000)^**^ | Denmark | 30,464 | 56.5 | 56 | 14.5 |  | 8,549 | NA^††^ | NA | 6 | +0 |  | √ |
| Chyou (2000) | USA | 367 | 72.0 | 100 | 9.0 |  | 30 | Serum | Yes | 5 | +3 |  | √ |
| Froom (2000) | Israel | 3,461 | 44.3 | 100 | 11.0 | 84 |  | Serum | No | 6 | +0 | √ |  |
| Kilander (2001) | Sweden | 2,285 | 50.1 | 100 | 25.7 | 301 | 630 | Serum | Yes | 6 | +4 | √ |  |
| Cheng (2002) | USA | 27,459 | 40.8 | 100 | 13.0 | 205 | 724 | Serum | Yes | 7 | +3 | √ |  |
| Henry (2002) | Netherlands | 631 | 64.0 | 48 | 10.2 | 50 | 117 | Serum | Yes | 6 | +0 | √^＃＃^ | √^＃＃^ |
| Lindquist (2002) | Sweden | 1,462 | 40.2 | 0 | 24.0 |  | 265 | Serum | Yes | 7 | +7 | √ |  |
| Eberly (2003) | USA | 2,809 | 46 | 100 | 25.0 | 328 |  | Serum | No | 8 | +2 | √^＃＃^ |  |
| Johansson (2003) | Sweden | 1,372 | 49.3 | 0 | 19.0 |  | 164 | Serum | Yes | 8 | +3 | √ | √ |
| He (2004) | China | 1,696 | 44.0 | 66 | 24.0 |  | 306 | Serum | Yes | 6 | +4 | √ |  |
| Juutilainen (2004) | Finland | 1296 | 54.6 | 45 | 13.0 | 43 |  | NA | NA | 8 | +2 | √^＃＃^ |  |
| Psaty (2004) | USA | 4,885 | 73.0 | 40 | 7.5 |  | 1,096 | Plasma | Yes | 7 | +1 | √ |  |
| APCSC (2004 )^♀^ | Six countries | 96,224 | 48.4 | 52 | 7.9 | 1,337 |  | Serum | NA | 7 | +0 |  | √ |
| APCSC（2005）^﹟^ | Seven countries | 331,100 | 47.0 | 59 | 4.0 | 2,082 |  | Serum | NA | 7 | +1 | √ |  |
| Baibas (2005) | Greece | 1,150 | 50.0 | 44 | 14.0 | 67 | 297 | Serum | NA | 6 | +3 | √ |  |
| Mazza (2005) | Italy | 2,854 | 73.8 | 38 | 12.0 | 122 |  | Serum | Yes | 7 | +3 | √ | √ |
| Ulmer (2005) | Austria | 44,649 | 51.0 | 43 | 10.0 | 487 |  | Serum | Yes | 6 | +3 | √^＃＃^ |  |
| Schupf (2005) | USA | 2,277 | 76.1 | 34 | 3.0 |  | 291 | Plasma | Yes | 5 | +6 |  | √ |
| Janssen (2005) | USA | 14,511 | 48.0 | 100 | 10.9 |  | 420 | Serum | Yes | 5 | +0 |  | √ |
| Liu (2005)^‖^ | USA | 18,363 | 47.1 | 65 | 13.0 | 659 |  | Plasma | Yes | 8 | +1 |  | √ |
| DECODE (2006)^&^ | Six countries | 10,269 | 50.0 | 46 | 10.6 | 299 |  | Serum | NA | 7 | +1 | √ |  |
| Forouhi (2006) | UK | 3207 | 52.0 | 100 | 16.2 | 108 |  | Plasma | Yes | 7 | +0 | √^＃＃^ |  |
| De Stavola (2007) | UK | 3,108 | 48.2 | 70 | 29.0 | 267 |  | NA | NA | 4 | +0 | √ | √ |
| Haheim (2007 ) | Norway | 14,403 | 45.4 | 100 | 21.0 | 485 |  | Serum | No | 8 | +3 | √ |  |
| Lan (2007) | China | 2,086 | 71.1 | 63 | 8.2 |  | 409 | Serum | Yes | 6 | +2 | √ |  |
| Sai (2007) | China | 1,239 | 63.0 | 100 | 18.0 |  | 491 | Plasma | NA | 7 | +3 | √ | √ |
| Shankar (2007 ) | Australia | 2,904 | 65.5 | 40 | 10.0 | 242 | 575 | Serum | Yes | 7 | +9 | √ | √ |
| Hsu (2008 ) | China | 11,058 | 49.5 | 44 | 10.6 | 246 | 942 | Serum | Yes | 5 | +0 | √ |  |
| Tsai (2008) | China | 3,5259 | 50.9 | 66 | 15.0 | 468 | 2,095 | Serum | Yes | 6 | +0 |  | √ |
| Mozaffarian (2008) | USA | 4,258 | 73.0 | 42 | 15.0 |  | 2,116 | Plasma | No | 8 | +5 |  | √ |
| Marshall (2008) | Australia | 380 | 73.0 | 53 | 13.3 |  | 33 | Serum | Yes | 5 | +0 | √ |  |
| Cesari (2009) | Italy | 336 | 85.5 | 33 | 2.0 |  | 86 | Serum | Yes | 5 | +6 | √^＃＃^ |  |
| Oterdoom (2009) | Netherlands | 3,432 | 49.0 | 45 | 7.0 |  | 130 | NA | Yes | 5 | +0 | √^＃＃^ |  |
| Upmeier (2009) | Finland | 877 | 70.0 | 36 | 12.0 | 252 | 316 | Serum | Yes | 8 | +2 |  | √ |
| Lindman (2010) | Norway | 86,261 | 39.5 | 51 | 27.0 | 4,916 | 14,795 | Serum | No | 8 | +3 | √ | √ |
| Holme (2011) | Norway | 14,846 | 44.5 | 100 | 33.0 |  | 6,904 | Serum | No | 7 | +0 | √^＃＃^ | √ |
| Langsted (2011) | Denmark | 13,972 | 54.0 | 46 | 30.0 | 2,049 | 8,814 | Plasma | No | 7 | +3 | √ |  |
| Werle (2011) | Brazil | 187 | 83.6 | 36 | 8.7 | 55 | 141 | NA | Yes | 5 | +0 |  | √ |
| Bae (2012) | Korea | 12,740 | 56.2 | 38 | 16.0 | 173 |  | Serum | No | 7 | +0 |  | √ |
| Hari (2012) | USA | 6,670 | 62.1 | 48 | 4.1 |  | 224 | Serum | Yes | 7 | +7 | √ |  |
| Sidorenkov (2012) | Russia | 3,704 | 45.0 | 53 | 10.2 | 129 | 242 | Serum | No | 6 | +3 |  |  |
| Lind (2012) | Sweden | 1,016 | 70.0 | 50 | 7.0 |  | 114 | Serum | Yes | 8 | +7 | √^＃＃^ |  |
| Nilsson (2013) | Sweden | 396 | 75.0 | 49 | 10.6 |  | 200 | Serum | Yes | 7 | +1 | √^＃＃^ |  |
| Oksala (2013) | Finland | 5956 | 46.3 | 49 | 7.8 | 55 |  | Serum | NA | 7 | +1 | √ |  |

† Mean or median age at baseline.

†† NA stand for mixed or unknown ones.

‡ Mean or median duration of follow-up.

§ Methodological quality of studies based on the Newcastle-Ottawa Quality Assessment Scale.

¶ ABCDEFG+n : A: age; B: gender; C: blood pressure including systolic blood pressure and/or diastolic blood pressure; D: body mass index; E: smoking; F: diabetes (DM); G: alcohol consumption. Example: Lindman (2010) adjusted for adjusted age, gender, total cholesterol, systolic blood pressure, smoking, body mass index, menopausal status, time since meal, and physical activity, but not for alcohol drinking, and diabetes. +n: number of other factors.

** There are three cohorts included in this study (CCHS: the Copenhagen City Heart Study; CMS: the Copenhagen Male Study; GPS: the Glostrup Population Studies)

TG was analyzed as a continuous or categorical variable

＃＃ TG was logarithmic transformation as a continuous variable.

‖There are four cohorts included in this study (FCS: Framingham Cohort Study; FOS: Framingham Offspring Study; LRCF: Lipid Research Clinics Prevalence Follow-up Study; MRFIT: Multiple Risk Factors Intervention Trial)

♀APCSC, the Asia Pacific Cohort Studies Collaboration, an individual participant data meta-analysis of 26 prospective studies in the Asia-Pacific region, TG was analyzed as a category variable

# APCSC, an individual participant data meta-analysis of 32 studies from the Asia-Pacific region, TG was analyzed as a continuous variable.

＆ An individual data of nine European cohort studies participating in the Diabetes Epidemiology: Collaborative Analysis Of Diagnostic Criteria in Europe (DECODE)

Reference

1. Pelkonen R, Nikkila EA, Koskinen S, Penttinen K, Sarna S: **Association of serum lipids and obesity with cardiovascular mortality.** *British medical journal* 1977, **2:**1185-1187.

2. Yano K, Reed DM, McGee DL: **Ten-year incidence of coronary heart disease in the Honolulu Heart Program. Relationship to biologic and lifestyle characteristics.** *Am J Epidemiol* 1984, **119:**653-666.

3. Eschwege E, Richard JL, Thibult N, Ducimetiere P, Warnet JM, Claude JR, Rosselin GE: **Coronary heart disease mortality in relation with diabetes, blood glucose and plasma insulin levels. The Paris Prospective Study, ten years later.** *Horm Metab Res Suppl* 1985, **15:**41-46.

4. Reed D, Yano K, Kagan A: **Lipids and lipoproteins as predictors of coronary heart disease, stroke, and cancer in the Honolulu Heart Program.** *Am J Med* 1986, **80:**871-878.

5. Barrett-Connor E, Khaw KT: **Borderline fasting hypertriglyceridemia: absence of excess risk of all-cause and cardiovascular disease mortality in healthy men without hypercholesterolemia.** *Preventive medicine* 1987, **16:**1-8.

6. Tverdal A, Foss OP, Leren P, Holme I, Lund-Larsen PG, Bjartveit K: **Serum triglycerides as an independent risk factor for death from coronary heart disease in middle-aged Norwegian men.** *Am J Epidemiol* 1989, **129:**458-465.

7. Cowan LD, O'Connell DL, Criqui MH, Barrett-Connor E, Bush TL, Wallace RB: **Cancer mortality and lipid and lipoprotein levels. Lipid Research Clinics Program Mortality Follow-up Study.** *Am J Epidemiol* 1990, **131:**468-482.

8. Menotti A, Spagnolo A, Scanga M, Dima F: **Multivariate prediction of coronary deaths in a 10 year follow-up of an Italian occupational male cohort.** *Acta Cardiol* 1992, **47:**311-320.

9. Haheim LL, Holme I, Hjermann I, Leren P: **The predictability of risk factors with respect to incidence and mortality of myocardial infarction and total mortality. A 12-year follow-up of the Oslo Study, Norway.** *J Intern Med* 1993, **234:**17-24.

10. Criqui MH, Heiss G, Cohn R, Cowan LD, Suchindran CM, Bangdiwala S, Kritchevsky S, Jacobs DR, Jr., O'Grady HK, Davis CE: **Plasma triglyceride level and mortality from coronary heart disease.** *N Engl J Med* 1993, **328:**1220-1225.

11. Stensvold I, Tverdal A, Urdal P, Graff-Iversen S: **Non-fasting serum triglyceride concentration and mortality from coronary heart disease and any cause in middle aged Norwegian women.** *Bmj* 1993, **307:**1318-1322.

12. Menotti A, Scanga M, Morisi G: **Serum triglycerides in the prediction of coronary artery disease (an Italian experience).** *Am J Cardiol* 1994, **73:**29-32.

13. Sahyoun NR, Jacques PF, Dallal G, Russell RM: **Use of albumin as a predictor of mortality in community dwelling and institutionalized elderly populations.** *J Clin Epidemiol* 1996, **49:**981-988.

14. Simons LA, McCallum J, Friedlander Y, Simons J: **Predictors of mortality in the prospective Dubbo study of Australian elderly.** *Australian and New Zealand journal of medicine* 1996, **26:**40-48.

15. Tunstall-Pedoe H, Woodward M, Tavendale R, A'Brook R, McCluskey MK: **Comparison of the prediction by 27 different factors of coronary heart disease and death in men and women of the Scottish Heart Health Study: cohort study.** *Bmj* 1997, **315:**722-729.

16. Vilbergsson S, Sigurdsson G, Sigvaldason H, Sigfusson N: **Coronary heart disease mortality amongst non-insulin-dependent diabetic subjects in Iceland: the independent effect of diabetes. The Reykjavik Study 17-year follow up.** *J Intern Med* 1998, **244:**309-316.

17. Bjornholt JV, Erikssen G, Aaser E, Sandvik L, Nitter-Hauge S, Jervell J, Erikssen J, Thaulow E: **Fasting blood glucose: an underestimated risk factor for cardiovascular death. Results from a 22-year follow-up of healthy nondiabetic men.** *Diabetes Care* 1999, **22:**45-49.

18. Andersen LB, Schnohr P, Schroll M, Hein HO: **All-cause mortality associated with physical activity during leisure time, work, sports, and cycling to work.** *Archives of internal medicine* 2000, **160:**1621-1628.

19. Chyou PH, Eaker ED: **Serum cholesterol concentrations and all-cause mortality in older people.** *Age and ageing* 2000, **29:**69-74.

20. Froom P, Kristal-Boneh E, Melamed S, Harari G, Benbassat J, Ribak J: **Serum total cholesterol and cardiovascular mortality in Israeli males: the CORDIS Study. Cardiovascular Occupational Risk Factor Determination in Israeli Industry.** *Isr Med Assoc J* 2000, **2:**668-671.

21. Kilander L, Berglund L, Boberg M, Vessby B, Lithell H: **Education, lifestyle factors and mortality from cardiovascular disease and cancer. A 25-year follow-up of Swedish 50-year-old men.** *International journal of epidemiology* 2001, **30:**1119-1126.

22. Cheng YJ, Macera CA, Church TS, Blair SN: **Heart rate reserve as a predictor of cardiovascular and all-cause mortality in men.** *Med Sci Sports Exerc* 2002, **34:**1873-1878.

23. Henry RM, Kostense PJ, Bos G, Dekker JM, Nijpels G, Heine RJ, Bouter LM, Stehouwer CD: **Mild renal insufficiency is associated with increased cardiovascular mortality: The Hoorn Study.** *Kidney Int* 2002, **62:**1402-1407.

24. Lindquist P, Bengtsson C, Lissner L, Bjorkelund C: **Cholesterol and triglyceride concentration as risk factors for myocardial infarction and death in women, with special reference to influence of age.** *J Intern Med* 2002, **251:**484-489.

25. Eberly LE, Stamler J, Neaton JD: **Relation of triglyceride levels, fasting and nonfasting, to fatal and nonfatal coronary heart disease.** *Archives of internal medicine* 2003, **163:**1077-1083.

26. Johansson S, Wilhelmsen L, Lappas G, Rosengren A: **High lipid levels and coronary disease in women in Goteborg--outcome and secular trends: a prospective 19 year follow-up in the BEDA*study.** *Eur Heart J* 2003, **24:**704-716.

27. He Y, Lam TH, Li LS, He SF, Liang BQ: **Triglyceride and coronary heart disease mortality in a 24-year follow-up study in Xi'an, China.** *Ann Epidemiol* 2004, **14:**1-7.

28. Juutilainen A, Kortelainen S, Lehto S, Ronnemaa T, Pyorala K, Laakso M: **Gender difference in the impact of type 2 diabetes on coronary heart disease risk.** *Diabetes Care* 2004, **27:**2898-2904.

29. Psaty BM, Anderson M, Kronmal RA, Tracy RP, Orchard T, Fried LP, Lumley T, Robbins J, Burke G, Newman AB, Furberg CD: **The association between lipid levels and the risks of incident myocardial infarction, stroke, and total mortality: The Cardiovascular Health Study.** *Journal of the American Geriatrics Society* 2004, **52:**1639-1647.

30. Collaboration APCS: **Serum triglycerides as a risk factor for cardiovascular diseases in the Asia-Pacific region.** *Circulation* 2004, **110:**2678-2686.

31. Collaboration APCS: **A comparison of the associations between risk factors and cardiovascular disease in Asia and Australasia.** *Eur J Cardiovasc Prev Rehabil* 2005, **12:**484-491.

32. Baibas N, Trichopoulou A, Voridis E, Trichopoulos D: **Residence in mountainous compared with lowland areas in relation to total and coronary mortality. A study in rural Greece.** *Journal of epidemiology and community health* 2005, **59:**274-278.

33. Mazza A, Tikhonoff V, Schiavon L, Casiglia E: **Triglycerides + high-density-lipoprotein-cholesterol dyslipidaemia, a coronary risk factor in elderly women: the CArdiovascular STudy in the ELderly.** *Intern Med J* 2005, **35:**604-610.

34. Ulmer H, Kollerits B, Kelleher C, Diem G, Concin H: **Predictive accuracy of the SCORE risk function for cardiovascular disease in clinical practice: a prospective evaluation of 44 649 Austrian men and women.** *Eur J Cardiovasc Prev Rehabil* 2005, **12:**433-441.

35. Schupf N, Costa R, Luchsinger J, Tang MX, Lee JH, Mayeux R: **Relationship between plasma lipids and all-cause mortality in nondemented elderly.** *Journal of the American Geriatrics Society* 2005, **53:**219-226.

36. Janssen I, Katzmarzyk PT, Church TS, Blair SN: **The Cooper Clinic Mortality Risk Index: clinical score sheet for men.** *American journal of preventive medicine* 2005, **29:**194-203.

37. Liu J, Sempos C, Donahue RP, Dorn J, Trevisan M, Grundy SM: **Joint distribution of non-HDL and LDL cholesterol and coronary heart disease risk prediction among individuals with and without diabetes.** *Diabetes Care* 2005, **28:**1916-1921.

38. The DECODE Study Group: **Comparison of different definitions of the metabolic syndrome in relation to cardiovascular mortality in European men and women.** *Diabetologia* 2006, **49:**2837-2846.

39. Forouhi NG, Sattar N, Tillin T, McKeigue PM, Chaturvedi N: **Do known risk factors explain the higher coronary heart disease mortality in South Asian compared with European men? Prospective follow-up of the Southall and Brent studies, UK.** *Diabetologia* 2006, **49:**2580-2588.

40. De Stavola BL, Meade TW: **Long-term effects of hemostatic variables on fatal coronary heart disease: 30-year results from the first prospective Northwick Park Heart Study (NPHS-I).** *J Thromb Haemost* 2007, **5:**461-471.

41. Haheim LL, Tonstad S, Hjermann I, Leren P, Holme I: **Predictiveness of body mass index for fatal coronary heart disease in men according to length of follow-up: a 21-year prospective cohort study.** *Scand J Public Health* 2007, **35:**4-10.

42. Lan TY, Chiu HC, Chang HY, Chang WC, Chen HY, Tai TY: **Clinical and laboratory predictors of all-cause mortality in older population.** *Arch Gerontol Geriatr* 2007, **45:**327-334.

43. Sai XY, He Y, Men K, Wang B, Huang JY, Shi QL, Zhang L, Li LS, Choi BC, Yan YP: **All-cause mortality and risk factors in a cohort of retired military male veterans, Xi'an, China: an 18-year follow up study.** *BMC Public Health* 2007, **7:**290.

44. Shankar A, Mitchell P, Rochtchina E, Wang JJ: **The association between circulating white blood cell count, triglyceride level and cardiovascular and all-cause mortality: population-based cohort study.** *Atherosclerosis* 2007, **192:**177-183.

45. Hsu PF, Chuang SY, Cheng HM, Tsai ST, Chou P, Chen CH: **Clinical significance of the metabolic syndrome in the absence of established hypertension and diabetes: A community-based study.** *Diabetes Res Clin Pract* 2008, **79:**461-467.

46. Tsai SP, Wen CP, Chan HT, Chiang PH, Tsai MK, Cheng TY: **The effects of pre-disease risk factors within metabolic syndrome on all-cause and cardiovascular disease mortality.** *Diabetes Res Clin Pract* 2008, **82:**148-156.

47. Mozaffarian D, Kamineni A, Prineas RJ, Siscovick DS: **Metabolic syndrome and mortality in older adults: the Cardiovascular Health Study.** *Archives of internal medicine* 2008, **168:**969-978.

48. Marshall NS, Wong KK, Liu PY, Cullen SR, Knuiman MW, Grunstein RR: **Sleep apnea as an independent risk factor for all-cause mortality: the Busselton Health Study.** *Sleep* 2008, **31:**1079-1085.

49. Cesari M, Onder G, Zamboni V, Capoluongo E, Russo A, Bernabei R, Pahor M, Landi F: **C-reactive protein and lipid parameters in older persons aged 80 years and older.** *J Nutr Health Aging* 2009, **13:**587-593.

50. Oterdoom LH, de Vries AP, van Ree RM, Gansevoort RT, van Son WJ, van der Heide JJ, Navis G, de Jong PE, Gans RO, Bakker SJ: **N-terminal pro-B-type natriuretic peptide and mortality in renal transplant recipients versus the general population.** *Transplantation* 2009, **87:**1562-1570.

51. Upmeier E, Lavonius S, Lehtonen A, Viitanen M, Isoaho H, Arve S: **Serum lipids and their association with mortality in the elderly: a prospective cohort study.** *Aging Clin Exp Res* 2009, **21:**424-430.

52. Lindman AS, Veierod MB, Tverdal A, Pedersen JI, Selmer R: **Nonfasting triglycerides and risk of cardiovascular death in men and women from the Norwegian Counties Study.** *Eur J Epidemiol* 2010, **25:**789-798.

53. Holme I, Tonstad S: **Risk factors and mortality--a follow-up of the Oslo Health Study from 1972-73.** *Tidsskrift for den Norske laegeforening : tidsskrift for praktisk medicin, ny raekke* 2011, **131:**456-460.

54. Langsted A, Freiberg JJ, Tybjaerg-Hansen A, Schnohr P, Jensen GB, Nordestgaard BG: **Nonfasting cholesterol and triglycerides and association with risk of myocardial infarction and total mortality: the Copenhagen City Heart Study with 31 years of follow-up.** *J Intern Med* 2011, **270:**65-75.

55. Werle MH, Moriguchi E, Fuchs SC, Bruscato NM, de Carli W, Fuchs FD: **Risk factors for cardiovascular disease in the very elderly: results of a cohort study in a city in southern Brazil.** *Eur J Cardiovasc Prev Rehabil* 2011, **18:**369-377.

56. Bae JM, Yang YJ, Li ZM, Ahn YO: **Low cholesterol is associated with mortality from cardiovascular diseases: a dynamic cohort study in Korean adults.** *J Korean Med Sci* 2012, **27:**58-63.

57. Hari P, Nerusu K, Veeranna V, Sudhakar R, Zalawadiya S, Ramesh K, Afonso L: **A gender-stratified comparative analysis of various definitions of metabolic syndrome and cardiovascular risk in a multiethnic U.S. population.** *Metab Syndr Relat Disord* 2012, **10:**47-55.

58. Sidorenkov O, Nilssen O, Grjibovski AM: **Determinants of Cardiovascular and All-Cause Mortality in Northwest Russia: A 10-Year Follow-Up Study.** *Annals of Epidemiology* 2012, **22:**57-65.

59. Lind L, Simon T, Johansson L, Kotti S, Hansen T, Machecourt J, Ninio E, Tedgui A, Danchin N, Ahlstrom H, Mallat Z: **Circulating levels of secretory- and lipoprotein-associated phospholipase A2 activities: relation to atherosclerotic plaques and future all-cause mortality.** *Eur Heart J* 2012, **33:**2946-2954.

60. Nilsson G, Hedberg P, Ohrvik J: **Inflammation and the metabolic syndrome: clustering and impact on survival in a Swedish community-based cohort of 75 year olds.** *Metab Syndr Relat Disord* 2013, **11:**92-101.

61. Oksala N, Seppala I, Hernesniemi J, Lyytikainen LP, Kahonen M, Makela KM, Reunanen A, Jula A, Ala-Korpela M, Lehtimaki T: **Complementary prediction of cardiovascular events by estimated apo- and lipoprotein concentrations in the working age population. The Health 2000 Study.** *Ann Med* 2013, **45:**141-148.
