# Supplementary figures and images for: Effects of blood triglycerides on cardiovascular and all-cause mortality: a systematic review and meta-analysis of 61 prospective studies
Source: Lipids Health Dis. 2013 Oct 29;12:159. doi: 10.1186/1476-511X-12-159 (PMC4231478; doi:10.1186/1476-511X-12-159)

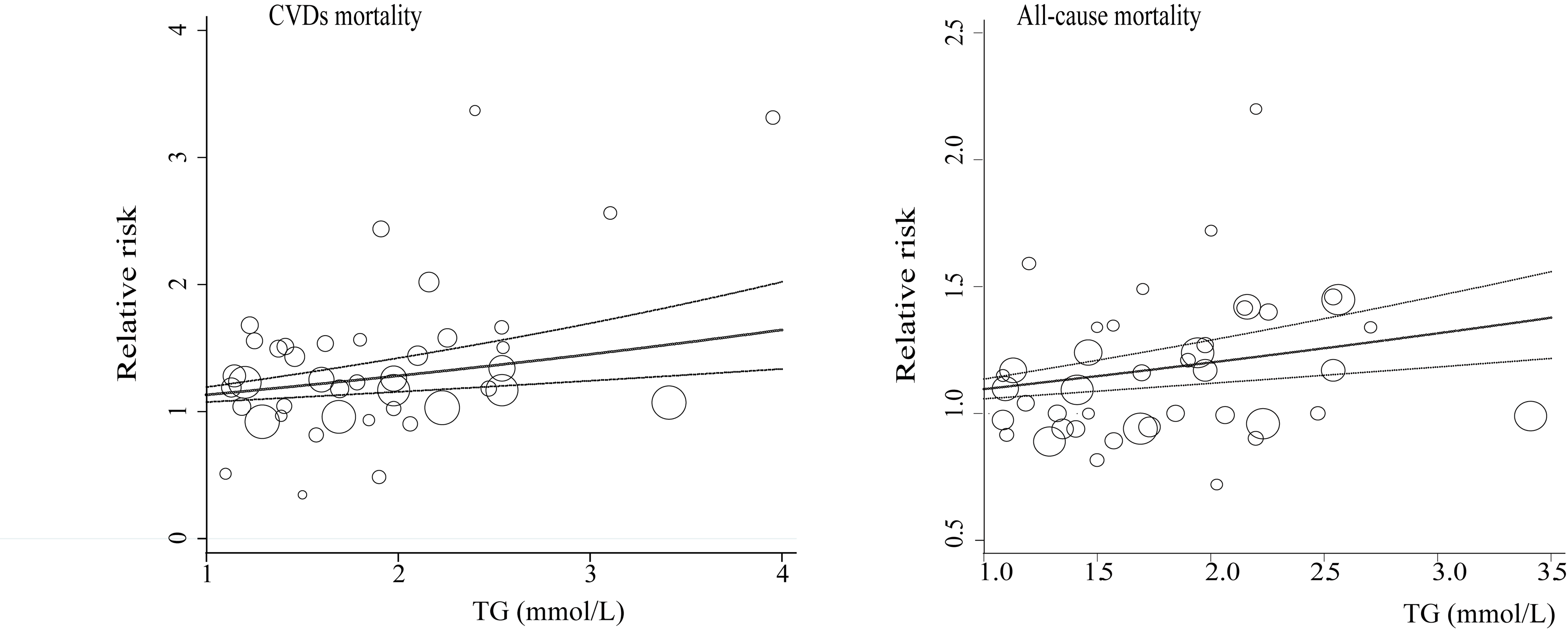

Supplement: Additional file 2: Figure S1 — Dose–response relationships between TG and risk of CVDs and all-cause mortality. The dots represent the RRs corresponding to TG levels in each individual study. The area of the dots is inversely proportional to the logarithm of the RR variance. The three curves are the RR estimates and their 95% CIs according to the dose–response model. CVDs, cardiovascular diseases; CI, confidence interval; RR, relative risk; TG, triglycerides. [file 1476-511X-12-159-S2.tiff]
