# Supplementary material for: Effects of blood triglycerides on cardiovascular and all-cause mortality: a systematic review and meta-analysis of 61 prospective studies
Source: Lipids Health Dis. 2013 Oct 29;12:159. doi: 10.1186/1476-511X-12-159 (PMC4231478; doi:10.1186/1476-511X-12-159)
Supplement: Additional file 3: Table S3 — Meta regression analysis of TG and CVDs and all-cause mortality for continuous analysis. [file 1476-511X-12-159-S3.doc]

**Additional file 3 Meta regression analysis of TG and CVDs and all-cause mortality for continuous analysis**

| Group | CVDs mortality | | | | All-cause mortality | | | |
| --- | --- | --- | --- | --- | --- | --- | --- | --- |
|  | β | SE | t | P>\|t\| | β | SE | t | P>\|t\| |
| Age (>50 vs ≤50 years) | 0.063 | 0.061 | 1.030 | 0.311 | 0.037 | 0.040 | 0.920 | 0.367 |
| Gender (women vs men/mix) | 0.116 | 0.047 | 2.450 | 0.020 | 0.077 | 0.027 | 2.880 | 0.008 |
| Follow-up (>15vs ≤15 years) | 0.068 | 0.061 | 1.110 | 0.276 | 0.109 | 0.033 | 3.290 | 0.003 |
| Fast status (fast vs non-fast/NA) | -0.029 | 0.041 | -0.700 | 0.491 | -0.010 | 0.035 | -0.280 | 0.781 |
| Geographic location  (Europe-America vs Asia-Pacific) | -0.165 | 0.106 | -1.550 | 0.132 | -0.018 | 0.052 | -0.340 | 0.735 |
| Quality score (>6 vs ≤6 scores) | -0.053 | 0.065 | -0.820 | 0.420 | -0.029 | 0.037 | -0.780 | 0.443 |
| Sample size (>4000 vs ≤4000) | 0.047 | 0.076 | 0.610 | 0.547 | -0.037 | 0.042 | -0.870 | 0.393 |
| Adjustment for TC (Yes vs No) | -0.131 | 0.059 | -2.23 | 0.034 | -0.014 | 0.041 | -0340 | 0.739 |
| Adjustment for HDL (Yes vs No) | -0.005 | 0.115 | -0.04 | 0.968 | -0.084 | 0.075 | -1.120 | 0.272 |
| Free of CVDs at baseline (Yes vs No) | -0.004 | 0.064 | 0.060 | 0.954 | -0.020 | 0.041 | -0.500 | 0.624 |

TG: triglycerides; TC: total cholesterol; CVDs: cardiovascular diseases; NA stand for mixed and unknown one
